# iSeizdiag: toward the framework development of epileptic seizure detection for healthcare

**DOI:** 10.3389/fncom.2025.1545425

**Published:** 2025-05-27

**Authors:** Ashish Sharma, Akshat Saxena, Mradul Agrawal, Kunal Kishor, Deepti Kaushik, Prateek Jain, Arvind R. Yadav, Manob Jyoti Saikia

**Affiliations:** ^1^Biomedical Sensors & Systems Lab, The University of Memphis, Memphis, TN, United States; ^2^Department of Computer Science and Engineering, Manipal University Jaipur, Jaipur, India; ^3^Department of Electronics and Communication Engineering, Indian Institute of Information Technology Kota, Kota, India; ^4^Department of Nursing, S R Goyal Government Hospital, SMS Medical College, Jaipur, India; ^5^Department of Electronics and Instrumentation Engineering, Nirma University, Ahmedabad, India; ^6^Electrical and Computer Engineering Department, The University of Memphis, Memphis, TN, United States

**Keywords:** EEG signal, temporospatial mapping, classification, epileptic, SVM, RF, KNN

## Abstract

**Introduction:**

The seizure episodes result from abnormal and excessive electrical discharges by a group of brain cells. EEG framework-based signal acquisition is the real-time module that records the electrical discharges produced by the brain cells. The electrical discharges are amplified and appear as a graph on electroencephalogram systems. Different neurological disorders are represented as different waves on EEG records.

**Method:**

This paper involves the detection of Epilepsy which appears as rapid spiking on electroencephalogram signals, using feature extraction and machine learning techniques. Various models, such as the Support Vector Machine, K Nearest Neighbor, and random forest, have been trained, and accuracy has been analyzed to predict the seizure.

**Result:**

An average accuracy of 95% has been claimed using the optimized model for epileptic seizure detection during training and validation. During the analysis of multiple models, the 97% accuracy is claimed after testing. Some statistical parameters are calculated to justify the optimized framework.

**Discussion:**

The proposed approach represents a satisfactory contribution in precise detection for smart healthcare.

## 1 Introduction

**Figure 1:**
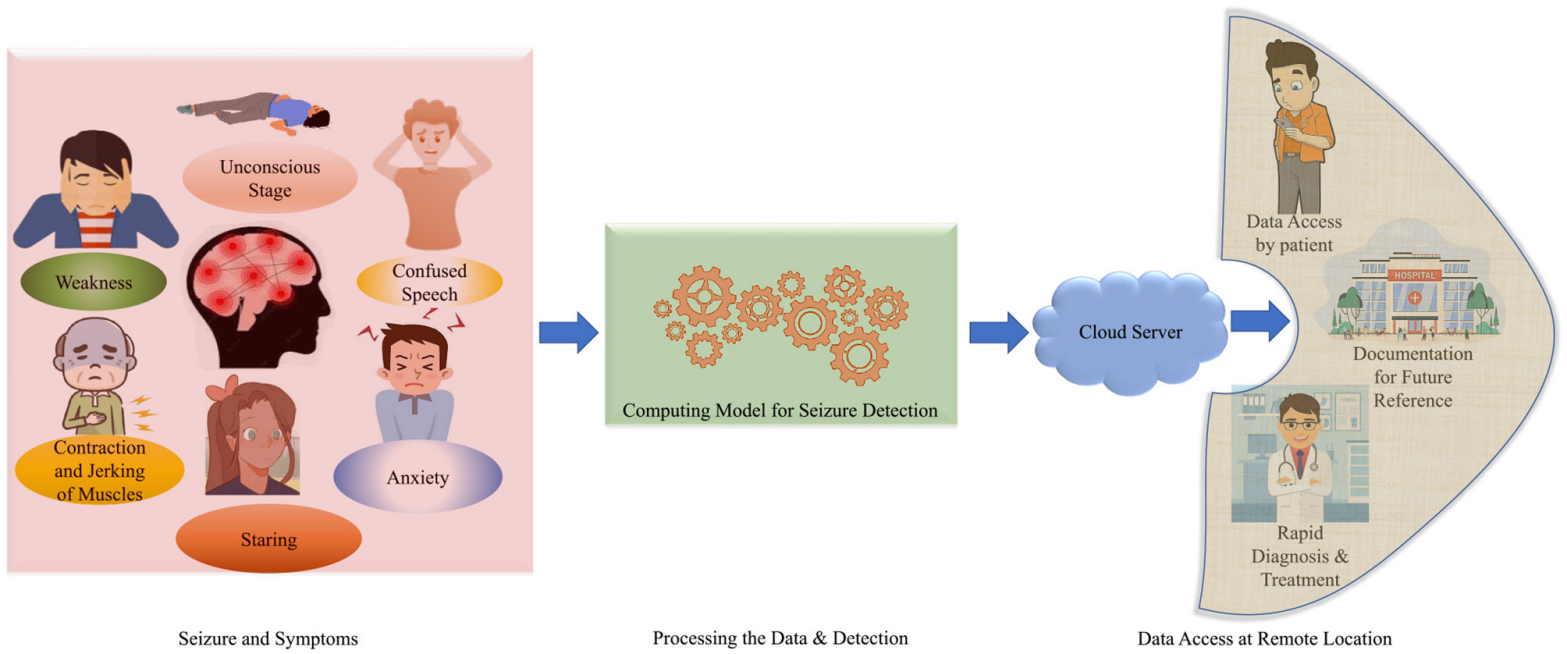
An overview of seizure symptoms, diagnosis and treatment for smart healthcare.

Epilepsy is a neurological disorder that affects the human brain. It is characterized by recurrent seizure episodes, which may include brief involvements of parts of the human or entire body. It is generally examined by abnormal and excessive brain cell activity, causing brief involuntary movements or shaking of the parts of the entire human body. These sudden involuntary movements can occur recurrently and are known as recurrent Seizures or Seizure episodes (Zheng et al., 2019). A person is said to be an Epilepsy patient if he experiences two or more unprovoked seizure episodes. Seizures can be life-threatening as a patient can experience physical trauma, like severe muscle jerks and prolonged convulsions as well as mental trauma like anxiety and depression (Park et al., 2019). They can affect a person's everyday life if not detected and treated immediately. The abnormal symptoms are visualized in Figure 1. About 50 million people worldwide have epilepsy, marking it as one of the most common neurological diseases. It also has been estimated that nearly 70% of epilepsy patients can live a seizure-free life if the disease is diagnosed and treated well in time. There are various traditional methods and advanced setups are available for proper diagnosis and better treatment. The traditional methods have been a lifeline for patients with neurological disorders for decades. With a global perspective of smart healthcare, the technologies are being advanced in terms of rapid diagnosis and better treatment (Olmi et al., 2021).

## 2 iSeizdiag is important for healthcare perspective

The traditional EEG systems are complex and require medical personnel to understand and identify the readings generated by the system to conclude whether the condition experienced by the patient qualifies as Epilepsy or any other neurological disorder (Yan et al., 2022). The medical representatives are required to analyze the problem and provide the proper treatment. This process is time-consuming and is not an affordable solution for everyone. Hence, it is required to conclude a precise and affordable setup for epileptic seizure detection (Olokodana et al., 2021). In this way, for rapid diagnosis and treatments; various advanced techniques and frameworks are required to be introduced to the patients in urban and remote areas. The data can be visualized and accessible at remote locations from the user and medical consultant end (Jain et al., 2019). The data can be stored as per the documentation for future access perspective (Joshi et al., 2020). This is reported in Figure 1.

**Figure 2:**
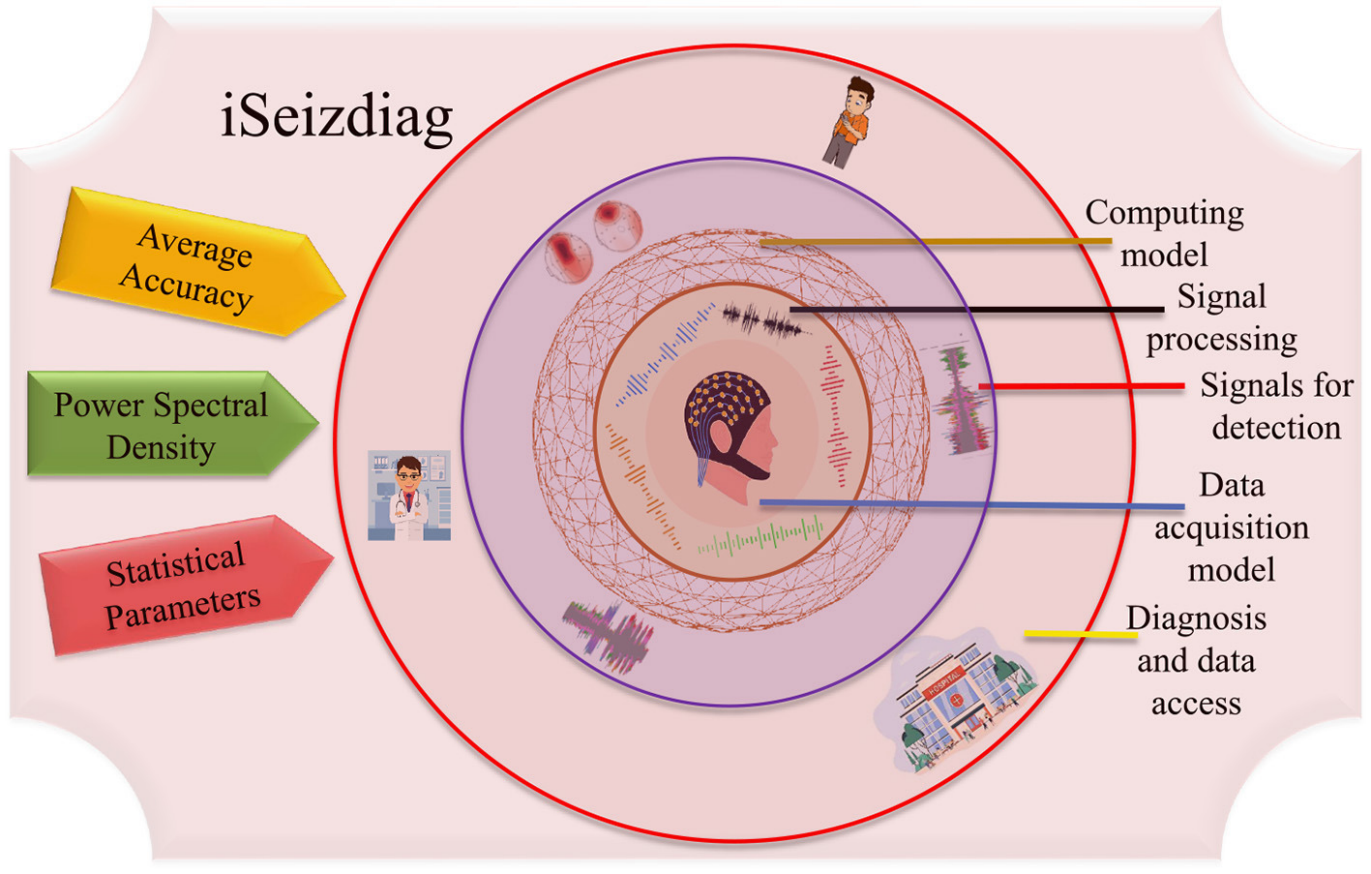
An overview of the proposed framework of iSeizdiag.

Therefore, It will be easier to use interpreTablebio-medical technologies and applications that are able to detect Epileptic Seizure episodes so that the patient can be brought to immediate medical attention. With the availability of sustainable and accurate methods that could stand along with the medical standards, there would be a relatively higher rate of success dealing with the critical cases of diseases such as Epilepsy (Yan et al., 2022). By deploying an advanced framework, the iSeizdiag would be an affordable solution in the consumer electronics paradigm (Olokodana et al., 2021). The proposed system would be a wearable system with higher accuracy and low power consumption (Sayeed et al., 2019a). The collected data will be accessible to the cloud server from authorized users (patients and doctors). The data can be stored for documentation and analysis perspective. The overview of the proposed framework is represented in Figure 2.

## 3 Related work

### 3.1 Signal processing paradigm for seizure detection

Signal processing techniques play a major role in the analysis of EEG signals before they can be used along with machine learning techniques to extract results from the EEG data. A thorough study of previous research involving EEG signals and the extensive use of signal processing techniques further solidifies their importance while working with EEG data (Pattnaik et al., 2022). A vast variety of studies have been done for the analysis and usage of physiological signals such as EEG etc. for the detailed study of neurological disorders like Epilepsy, Schizophrenia, Chronic Stress, Depression, Parkinson's disease, and Alzheimer's disease. Apart from that, many studies have analyzed EEG data with respect to the various types of human emotions and the associated brain cell activity.

### 3.2 Available dataset and prior proposed model on epileptic patients

The study was focused on detecting seizure episodes in epileptic patients (Gupta et al., 2020). The database acquired from Bonn University was used with 100 single-channel recordings sampled at a frequency of 173.6 Hz (Andrzejak et al., 2001). The accuracy is claimed to be 92% for seizure detection using smart headbands (Lin et al., 2018). Automatic epileptic seizure detection is introduced using EEG signals (Li et al., 2017). They proposed classification from time-frequency analysis using the radial basis function. Wang et al. (2018) represented accurate automatic seizure detection using wavelet decomposition and the directed transfer function (DTF) algorithm. The average sensitivity and detection are claimed as 92.1% and 95%, respectively. Gupta et al. (2018) explored the signal modeling technique for the classification of seizure and normal EEG signals. They claimed 95% average accuracy during analysis. An automated epileptic EEG detection system is proposed using iterative filtering (Sharma et al., 2018). 96% average accuracy is claimed using multiple classifier models. Yuan et al. explained EEG seizure detection using a multi-view deep learning framework with 94% classification accuracy (Yuan et al., 2018). Sayeed et al. (2019b) enlighten Neuro-Detect, which represents a fast and accurate seizure detection system for smart healthcare. Li et al. (2020) represented an intelligent recognition of epileptic EEG signals using a unified temporal-spectral squeeze and excitation block. The proposed framework indicated its powerful capability for automatic seizure detection. Sharma and Joshi (2021) proposed a novel approach to the detection of schizophrenia by using EEG data from two publicly available databases. The system is claimed to have resultant accuracy over 99%. EEG signals are analyzed for seizure detection using a multi-feature fusion approach (Radman et al., 2020). An optimized neural network is reported as a computing model for seizure detection. The proposed model is explained satisfactorily as a model for low-power wearable and implanTabledevices (Zhao et al., 2021). Other studies on epilepsy detection used databases that are publicly available on the internet. One such study was conducted by Shoeb (2009), and the data was collected from Children's Hospital Boston (Goldberger et al., 2000).

### 3.3 Problem addressed and possible solution discussed as per the prior work

There have also been a number of studies conducted on the use of EEG data for the analysis and detection of neurological disorders other than epilepsy and associated seizures. Support Vector Machine (SVM) was used as the classifier on the given data. Minimum negative valence and maximum arousal (NVHA) vs. Relax (R) and maximum valence and maximum arousal (HVHA) vs. relax (R) were the two trials in the focus in this study. A clear-cut study of emotion and its relationship with the hemispheres of the brain is prepared which helped us in analyzing the region of signal emittance for a particular thought generated. This diverse amount of work done on EEG signals, their ability to efficiently and effectively point out numerous neurological diseases, and the growing usage of electroencephalograms in the field of smart healthcare provide the major motivation for this work. The readily accessible and huge data repositories also provide the large amounts of data that are required for applications involving EEG signals. The need for a better understanding of the EEG activity of the human brain provides further motivation to implement visualization methods like channel-wise data plots and topographical maps to provide necessary insights into EEG data and the human brain. The prior works reported good results with different methodologies, which motivated the advancement of technologies for rapid diagnosis and treatment. The paper has been organized in the following manner: Section 4 is representing the highlights of the proposed work. Sections 5, 6 represent the problems and challenges of current objectives and the novel contribution of the proposed work respectively. Section 7 explained the proposed work for an epileptic seizure. Section 8 represents the conclusion and future direction of the proposed work.

## 4 Highlights of the current work

- Precise seizure detection has been confirmed using temporal-spatial mapping analysis.
- Classifier analysis has been done to justify an optimized computing model.
- DWT signals have been conditioned and visualized with data segmentation.
- Different statistical parameters are determined for DWT signals during the processing of the EEG signals.
- Accuracy analysis has been done to validate the diagnosis framework.

## 5 Merits and recent problems of epileptic seizure detection framework

Artifacts are very common physical constraints for creative false-positive seizure detection. To overcome this issue, a lots of framework has been proposed for precision points of view. As per the technical specification, it is to improve or enhance the signal quality, and durability without interference of the experts or medical representatives. Various techniques are proposed to optimize the framework to overcome these issues. These techniques helped diagnose the problems on time (Ahmad et al., 2024). Many researchers have explored wearable solutions with optimized computing models. However, it is required to design a system with a low-cost solution, that approaches minimum resources for manufacturing and diagnosis. Due to different motivational works on the EEG framework for smart healthcare, the current work is enlightened with a distinct optimized framework with novel features that represent statistical parameters and classification accuracy at the desired level (Anita and Kowshalya, 2024). The data can be accessed and manipulated by users and medical experts for rapid diagnosis and treatment.

## 6 Novel contribution in the current work

This work extends the existing methods of EEG Data and signal processing on the database provided by Children's Hospital Boston, MIT. The contribution in the present work is the judiciary explored in terms of advancement. The novel contribution is presented as working on creating a sustainable system for the detection of epilepsy. We have the following contributions summarizing a successful model for disease detection:

1. The implementation of this work allows the user to fetch the inter-ictal and ictal data of as many patients as desired.
2. Showcasing the differences between Inter-Ictal and Ictal data of Epilepsy patients by providing visual insights using channel data plots and topomaps for the EEG data.
3. The paper represents a unique framework for the EEG signal-based detection of Epileptic Seizure. The framework has incorporated unique processing methods for using EEG data and the validation and verification of the framework is supported by its high precision. Such a high-precision framework can contribute to the future development of high-precision EEG-based devices for consumers.
4. Visualizing topomaps can prove to be highly useful for studying and analyzing the patterns of EEG signals generated in diverse sets of neurological disorders. Topomap visualization is included in this study as the topomaps clearly illustrate why frequency-based methods like Fourier or Discrete wavelet transforms are highly effective in processing EEG data and differentiating between the information conveyed by EEG signals.

## 7 Proposed framework to detect epileptic seizure

**Figure 3:**
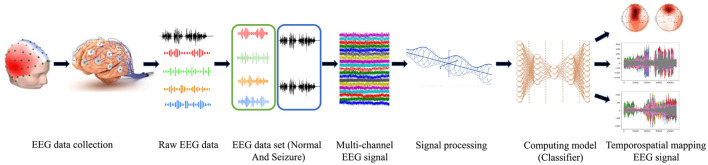
Processing steps for epileptic seizure detection.

An electroencephalogram captures brain signals with prior standards of data collection. The signals are processed and considered as raw EEG data. The raw EEG signals are a mixed form of normal and seizure data. The collected data in the form of signals is taken as a multi-channel EEG signal. The raw signals are filtered using an optimized window for data segmentation. The feature extraction is done using the wavelet transform method (Wang et al., 2018). The extracted signals are applied to train the classifier. The trained model is tested by testing data following testing standards for model validation. The output images are extracted for analysis and seizure detection. The processing framework is represented in Figure 3.

### 7.1 Description of dataset and processing

**Figure 4:**
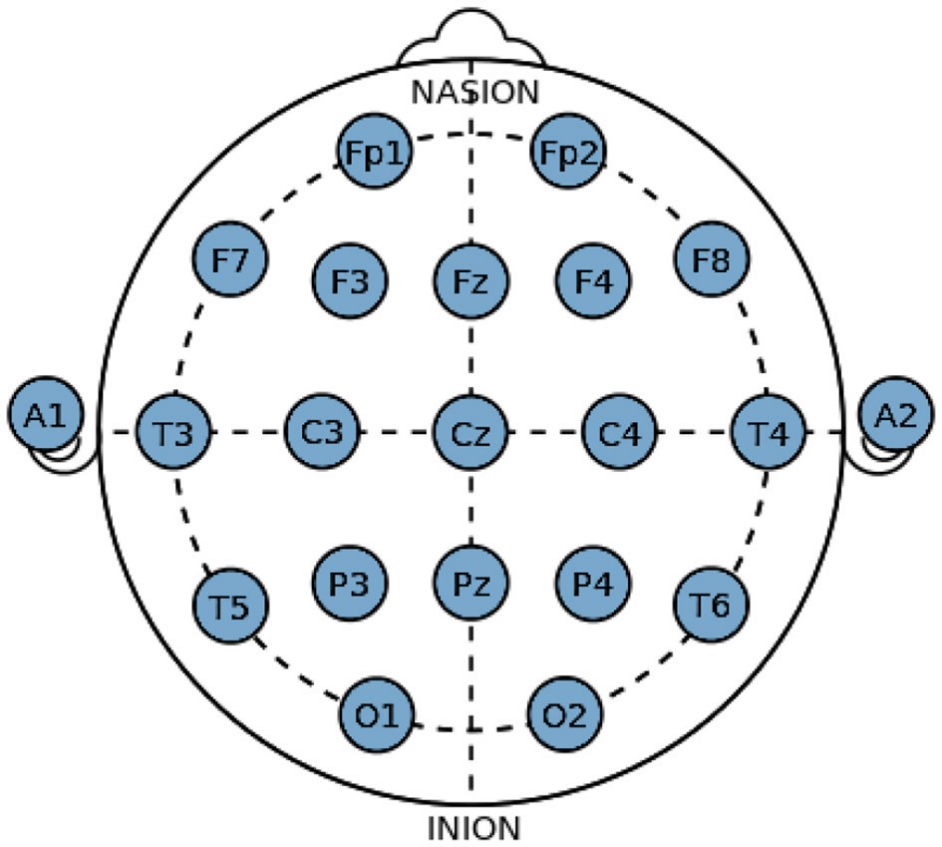
International 10–20 system for electrode placement.

EEG signals can be captured using invasive as well as non-invasive methods where the latter is generally considered a safer option. Due to the safety benefits, they bring along Non-invasive methods, which may give irregular results, but with proper filtering and electrode placement on the head. It can produce results similar to the invasive method. The non-invasive method requires a 10–20 Electrode Placement System to fulfill its purpose successfully. 10–20 electrode placement system is an international system used for placement of EEG electrodes on the scalp of a human (Sayeed et al., 2019b). This defines the locations of the different brain areas, as depicted in the Figure 4, which relate to the placement of electrodes, making a close relationship that stays the same for all the research based on EEG technology. Alphabets are assigned to mark the different locations of the lobes and a number to determine the hemispherical region of the brain. F, T, C, P, and O respectively stand for frontal, temporal, central, arietal, and occipital lobes, where the central is not a lobe but just an area to plot the other lobes correctly. Odd numbers (1, 3, 5, 7) define the left hemisphere of the brain while even number (2, 4, 6, 8) defines the right hemisphere (Zhao et al., 2021).

A vast archive of EEG data on epilepsy and Epileptic Seizures is available online. The dataset consists of invasive EEG Recordings of 21 patients suffering from medically intracTablefocal epilepsy (EEG Data, 2022). The recordings were taken during pre-surgical epileptic monitoring at the Epilepsy Center of the University of Hospital, Freiburg, Germany. The data consist of 24-h recordings acquired through a 128-channel, EEG acquisition system sampled at 256 Hz sampling frequency. For each patient, the data available are classified as ictal and interictal. The proposed dataset consists of 100 single-channel EEG recordings, sampled at 173.61 Hz (Andrzejak et al., 2001). The duration of each recording is about 23.6 seconds acquired through a 128-channel acquisition system. The dataset is divided into five sections: A, B, C, D, and E, where sections A and B consist of surface EEG recording from healthy subjects, sections C and D consist of intracranial EEG recording data recorded during seizure-free events from within and outside the seizure generating areas of the brain from epileptic patients. Section E is completely dedicated toward the EEG recording recorded during seizure episodes in epileptic patients. The dataset focused on in this proposed research work is the CHB-MIT Scalp EEG Database because of the vast amount of multi-channel data collected from the patients by using a non-invasive scalp EEG recording technique. A different dataset was collected at the Children's Hospital Boston and consists of EEG recordings from 22 pediatric patients (5 Males and 17 Females) with ages ranging from 1.5 to 19 years (EEG Data, 2022). The data includes 24 h of EEG recordings for each of the 22 patients. The international 10–20 System of EEG electrode positioning was used for the recordings. The data was recorded using an EEG acquisition system consisting of about 23 electrodes at a sampling frequency of 256 Hz. A total of 200 seizure events were recorded and are present in the database. The database also contains summary and annotation files, describing the exact start and end times of the EEG recording, including the epileptic seizure episodes.

### 7.2 Data preparation and filtering approach for classification

The database was accessed using PhysioNet (EEG Data, 2022). PhysioNet offers free access via the web to large collections of recorded physiological signals and related open-source software. All the file names were fetched using a package of PhysioNet itself, named “WFDB.” The waveform database (WFDB) package for Python is a library of tools for reading, writing, and processing physiological signals and annotations. The file-wise seizure windows were fetched from the summary file provided with the database and all the common channels were taken into a single array. Using these seizure windows, all the data from seizure files was concatenated vertically into a single array, and the equivalent amount (in seconds) of non-seizure data was fetched and concatenated vertically into a different array. Further, these data tables were categorized as seizure data and non-seizure data. Plotting was done on both segments channel-wise, and the plots were distinguishable. A finite impulse response filter is used to extract the signals at specific frequency levels. The impulse response is finite because there is no feedback in the filter. Here, the filter is used to eliminate noise at higher frequencies than that of EEG signals According to the Nyquist frequency theorem, a system used for signal acquisition at a sampling frequency of “s” can accurately record signals only up to the frequency of “s”/2, which is known as the Nyquist frequency. The EEG data recorded in the CHB-MIT database was sampled at 256 Hz and thus it was important to filter off any frequency components higher than 60 Hz present in the database, as most of the event-related potentials in the EEG data occur below the frequency of 60 Hz. Also, any components lower than 0.5 Hz also needed to be cut off so as to filter off the frequency components generated by the subject due to breathing, eye movements, etc.

### 7.3 Feature evaluation and extraction for detection

The most important and challenging aspect of working with EEG Data is extracting relevant features from the raw EEG data acquired using the acquisition system. The most popular techniques used to extract features from EEG data are the Power Spectral Density method using the Fourier Transform and the discrete wavelet transform. Power spectra are estimated by dividing the time signal into successive blocks, forming the periodogram for each block, and averaging. Denote the *t*_*t*_*h* windowed frame with zero-padding from the signal x in given Equation 1


(1) $$\begin{array}{l} x_{t}\left(p\right)\overset{\Delta }{=}w\left(p\right)x\left(p+tR\right);p=0,1,..P-1,t=0,1,..T-1 \end{array}$$

Where R is defined as the window hop size, and T represents the number of available frames. Then the periodogram of the *t*_*th*_ block is given in Equations 2, 3.


(2) $$\begin{array}{l} D_{xt},T\left(w_{p}\right)=\frac{1}{P}|FFT_{N,T}\left(x_{t}\right){|}^{2} \end{array}$$

and


(3) $$D_{xt},T(w_{p})\overset{\Delta }{=}\frac{1}{P}{\left|\sum_{p=0}^{P-1}x_{t}(p)-e^{\left(\frac{-j2\Pi pT}{P}\right)}\right|}^{2}$$

as before, and the Welch estimate of the power spectral density is given in Equation 4.


(4) $$\begin{array}{l} S_{x}^{W}\left(W_{T}\right)\overset{\overset{\Delta }{=}}{=}\frac{1}{T}\overset{T-1}{\underset{t=0}{\sum }}P_{xt},T\left(W_{T}\right) \end{array}$$

This is the average periodogram across time when w(p) is the rectangular window; the periodogram is formed from non-overlapping successive blocks of signal.

The Power Spectral Density method also known as Welch's periodogram, uses the Fourier Transform to calculate the power associated with the frequency components globally associated with the signals, i.e., frequencies persisting over the entire signal (Sayeed et al., 2019a). The periodograms were calculated and plotted for both categories of data. Fourier transformation provides a representation of the signal in the frequency domain; however, it fails to provide any kind of temporal information regarding the frequency components existing in the signal. That is why a better approach toward extracting features from raw EEG data is by using Discrete Wavelet Transformation.

The key advantage of using discrete wavelet transformations for feature extraction is their ability to provide both local spectral and temporal information simultaneously. The objective while performing wavelet decomposition using discrete wavelet transformation is to decompose the signals into a set of frequency components corresponding to bandwidth associated with different types of brainwaves: delta (0–4 Hz), theta (4–8 Hz), alpha (8–16 Hz), beta (16–32 Hz), and gamma (>32 Hz).

The EEG signal data of both categories was broken down into 2-second segments, or “Epochs,” containing 512 samples each. Then a wavelet decomposition using Daubechies “db4” Wavelet was applied to generate the corresponding set of wavelet coefficients for each of the channels. These coefficients were then used for calculating features like root mean square, mean, standard deviation, and skewness, generating a total of 20 × 18 features.

A signal *f*_*in*_ has an even number r of values, then the 1-level Db4 transform is the mapping of *f*_*in*_, which is *f*↦(*c*^1^∣*d*^1^) from the signal *f*_*in*_ to its first trend sub-signal *c*^1^ and first fluctuation sub-signal *d*^1^. Each value *c*_*q*_ of $c^{1}=\left(l_{1},l_{2},,,,,,l_{r/2}\right)$ is equal to a scalar product, in Equation 5


(5) $$\begin{array}{l} l_{q}=f.Y_{q}^{1} \end{array}$$

of f with a 1-level scaling signal $Y_{q}^{1}$. Similarly, each value *d*_*q*_ of *d*_*q*_ of $d^{1}=\left(d_{1},d_{2},,,,,,d_{r/2}\right)$ is equal to a scalar product in Equation 6.


(6) $$\begin{array}{l} d_{q}=f.S_{q}^{1} \end{array}$$

of f with a 1-level wavelet $S_{q}^{1}$. The 2nd level Daub4 scaling signals are presented by repeating the operations that were used on the natural basis of signals $Y_{1}^{0},Y_{2}^{0},Y_{3}^{0},Y_{4}^{0},...,Y_{r}^{0}$ to generate the first level scaling signals. Using this natural basis, the first-level Daub4 scaling signals satisfy (Equation 7).


(7) $$\begin{array}{l} Y_{q}^{1}=b_{1}.Y_{2q-1}^{0}+b_{2}.Y_{2q}^{0}+b_{3}.Y_{2q+1}^{0}+b_{4}.Y_{2q+2}^{0} \end{array}$$

with a wrap-around defined by $Y_{n+r}^{0}=Y_{n}^{0}$. Similarly, the second-level Daub4 scaling signals are defined by (Equation 8).


(8) $$\begin{array}{l} Y_{q}^{2}=b_{1}.Y_{2q-1}^{1}+b_{2}.Y_{2q}^{1}+b_{3}.Y_{2q+1}^{1}+b_{4}.Y_{2q+2}^{1} \end{array}$$

The wrap-around is defined by $Y_{n+r/2}^{1}=Y_{n}^{1}$.

This wraparound, or periodicity, of the first-level scaling signals is implied by the wrap-around invoked above for the natural signal basis.

### 7.4 Parametric evaluation and result analysis

To train, validate, and test the optimized model, statistical parameters have been considered. The parametric evaluation has been done using presented equation. The calculated values are analyzed to justify the compatible computing model. The expressions of parameters are as follows:

**Mean**: For some data, the arithmetic mean is the measure of the central tendency of a finite set of numbers. It is represented in Equation 9.


(9) $$\begin{array}{l} \bar{x}=\frac{1}{n}\left(\overset{n}{\underset{i=1}{\sum }}x_{i}\right) \end{array}$$

**Standard deviation**: For some given data, the standard deviation is a measure of the amount of variation or dispersion of a set of values. It is represented in Equation 10.


(10) $$\sigma =\sqrt{\frac{1}{N}\sum_{i=1}^{N}(x_{i}-\mu {)}^{2}}$$

**Root mean square**: It is also represented in Equation 11.


(11) $$\begin{array}{l} x_{RMS}=\sqrt{1/n\left(\overset{n}{\underset{i=1}{\sum }}x_{i}^{2}\right)} \end{array}$$

**Skewness**: Skewness is a measure of the asymmetry of the probability distribution of a real-valued random variable about its mean. It is represented in Equation 12.


(12) $$skewness=\frac{{\sum_{1}^{N}\left(x_{i}-\bar{x}\right)}^{3}}{(N-1)s^{3}}$$

**Kurtosis**: In statistics, kurtosis is used for measuring outliers in the data. It is represented in Equation 13.


(13) $$kurtosis=\frac{{\sum_{1}^{N}\left(x_{i}-\bar{x}\right)}^{4}}{(N-1)s^{4}}$$

**Figure 5:**
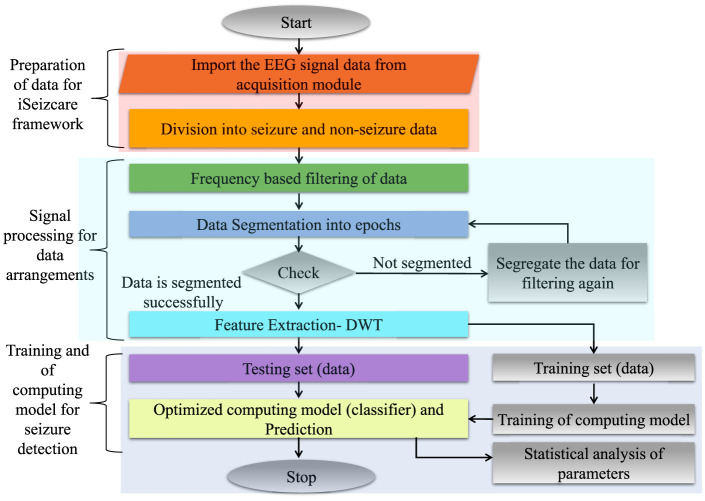
Block representation of pseudo-code for iSeizdiag.

**Figure 6:**
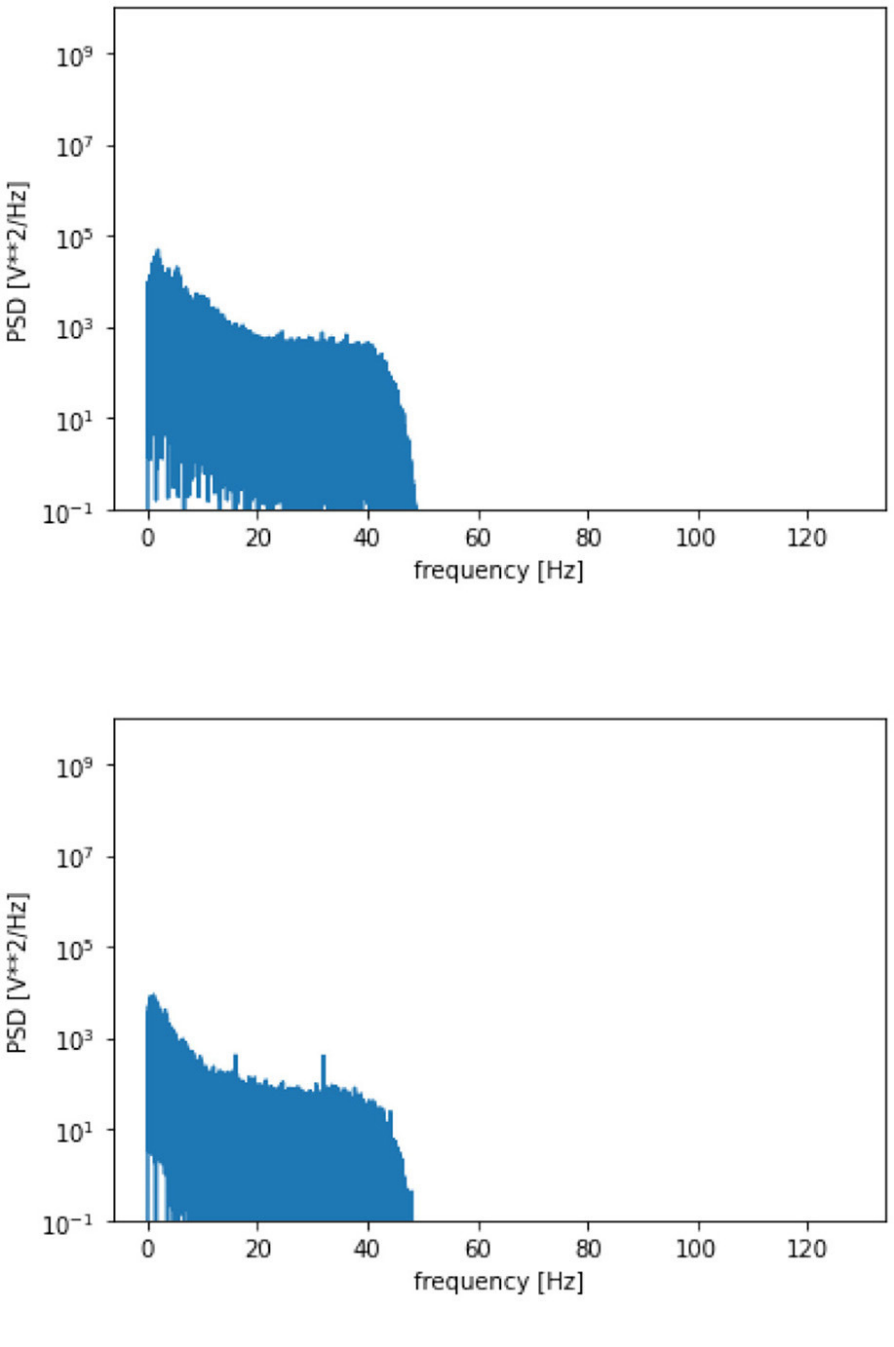
Power spectral density vs. frequency.

The proposed framework is introduced with certain standard steps, which represent the processing of raw data to diagnose the disease. The methodology is represented in Algorithm 1. The calculated parameters are considered to validate the framework for seizure detection. The preparation of the data, processing and training of the model have been done in a synchronization way. The processing flow with all necessary steps is represented in Figure 5. The calculated power spectra density with respect to frequency is visualized in Figure 6.

**Algorithm 1:**
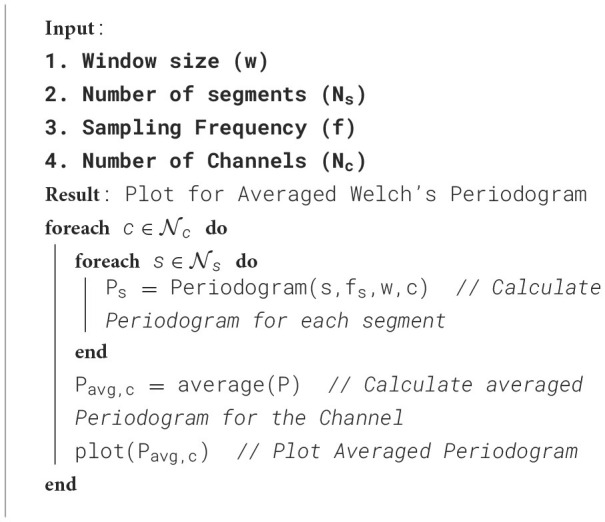
Plotting topomops using Welch's periodogram.

**Figure 7:**
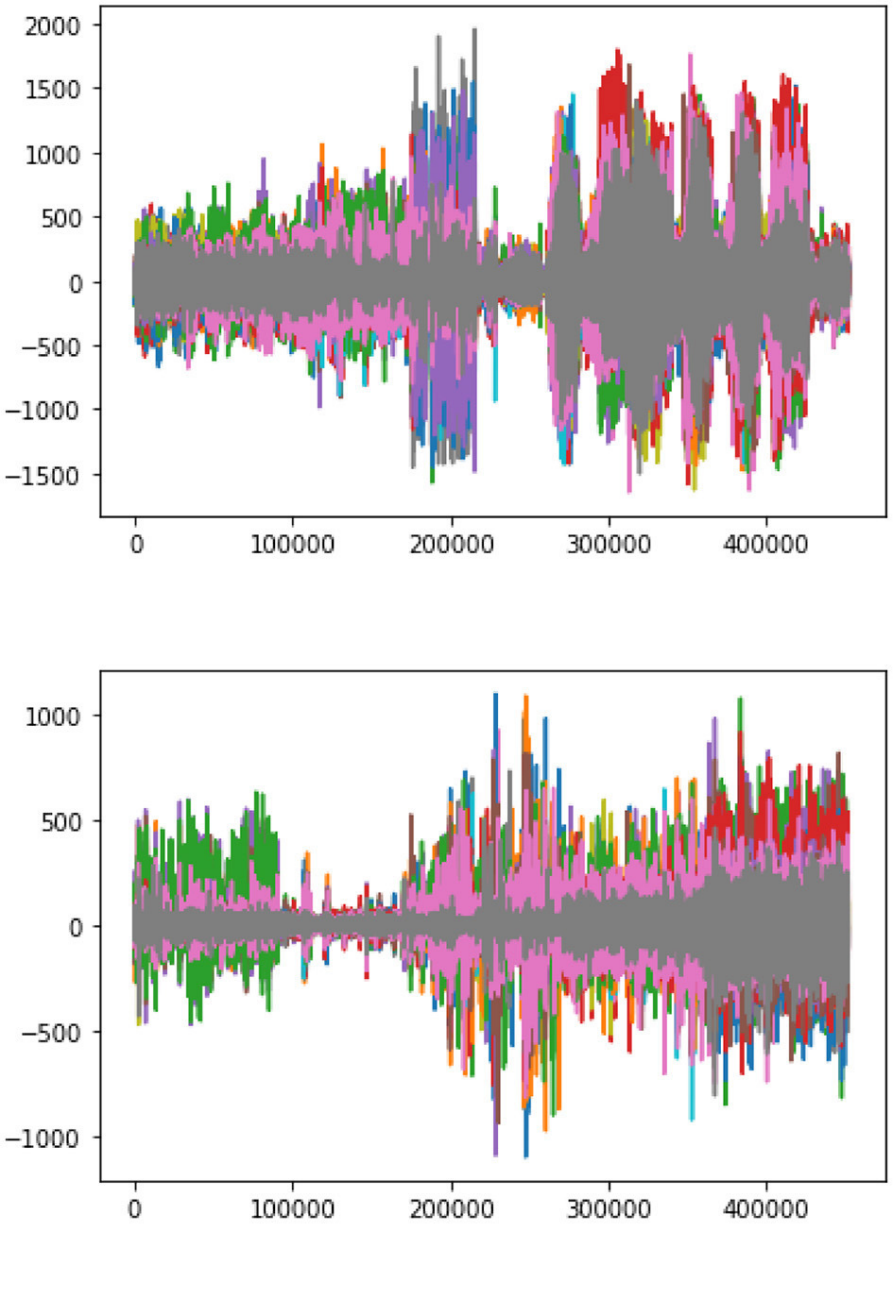
Amplitude vs. number of samples.

**Figure 8:**
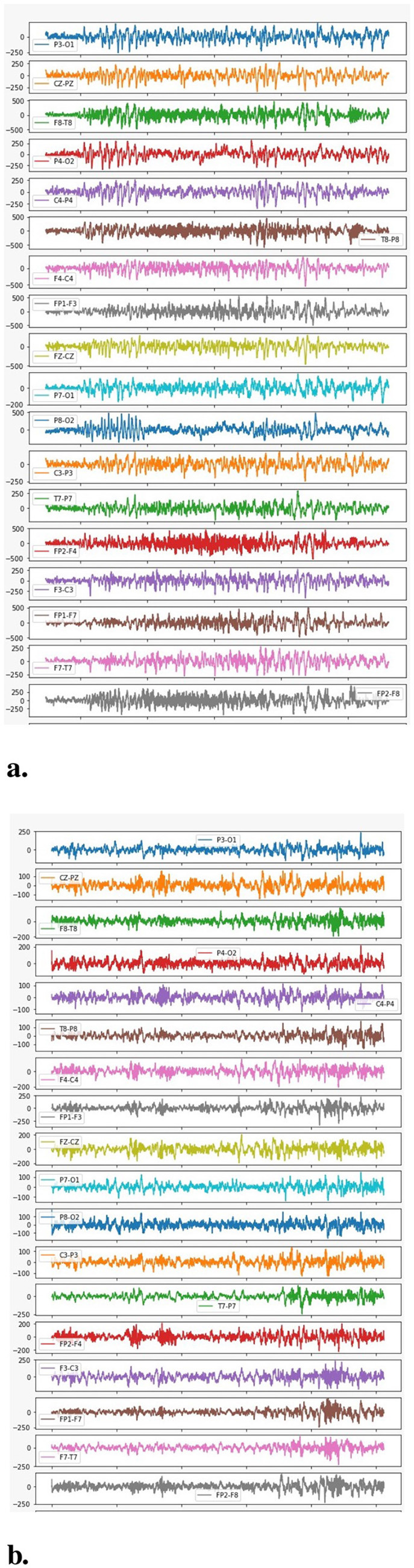
Amplitude vs. data samples. **(A)** Channel-wise plotting of Seizure samples. **(B)** Channel-wise plotting of Non-seizure samples.

The plotting between the EEG Data samples collected from multiple channels and their respective amplitude is presented in Figure 7. It clearly indicates that the EEG signals generated during a seizure period/Ictal period in an epileptic patient are higher in amplitude as compared to the signals generated during a non-seizure or Inter-ictal period. It is indicated by higher spikes in the plottings for seizure data. Similar results are represented in the individual plots of the channels plotted between EEG data samples and the respective amplitude for both seizure and non-seizure data. These are also explained in Figure 8B.

A more detailed analysis has also been performed by plotting the graphs between the frequency of the collected signals and their respective power spectral density as represented in Figure 8. The figure clearly indicates that higher EEG activity is generated in the lower frequency bands during a seizure and also, the general levels of EEG activity during a seizure period are higher than the EEG activity generated during a non-seizure period.

**Figure 9:**
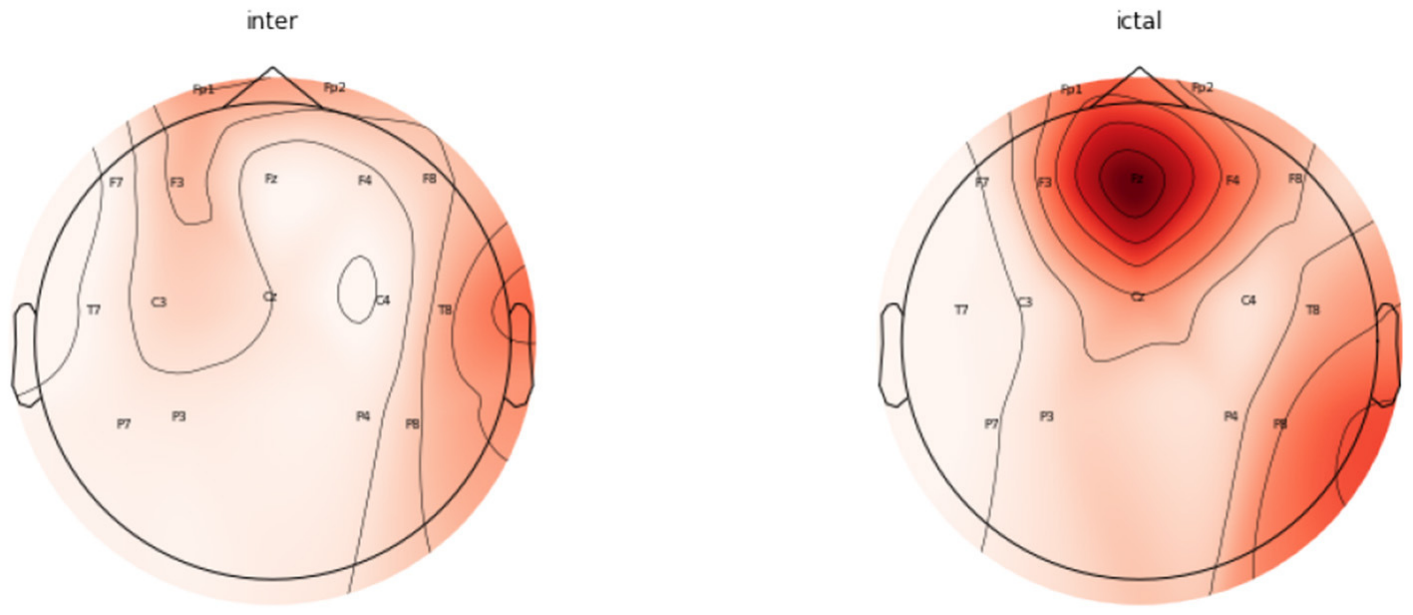
Topomaps for an epileptic patient for a duration of 30 ms during Inter-Ictal and Ictal Period.

**Figure 10:**
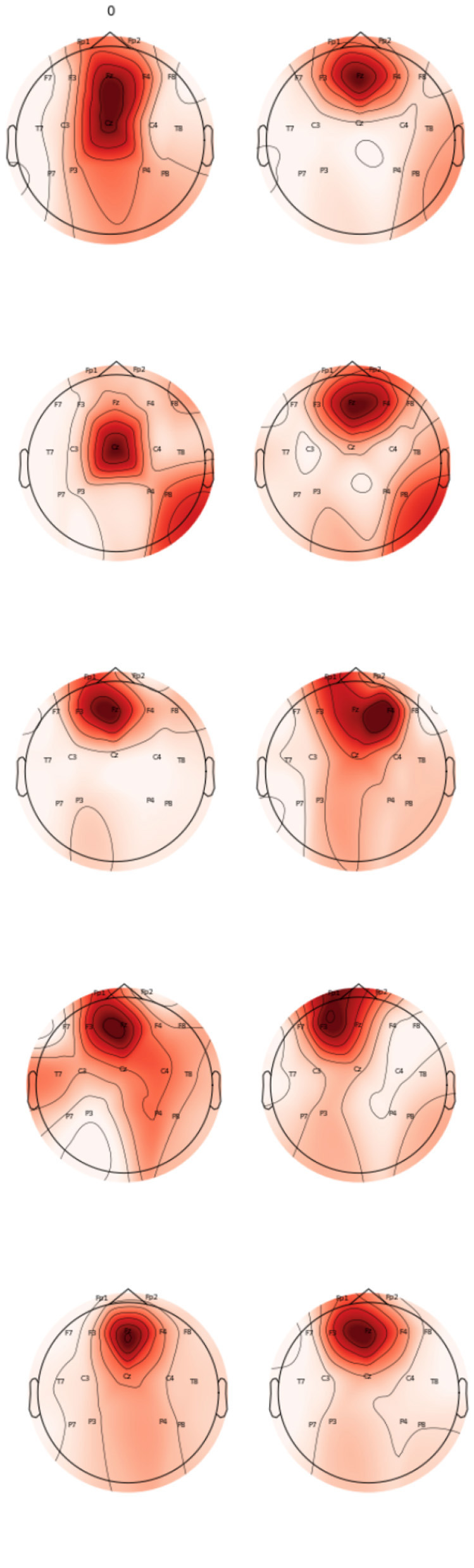
Topomaps for EEG activity spread over approx 95 ms.

The topomaps generated using power spectral density also confirm the above findings. Figure 9 represents the topomaps of an epileptic patient-generated during the Inter-ictal and Ictal period. It is again clearly evident that higher EEG activity is generated during the Ictal period as compared to the EEG activity generated during the Inter-ictal period. Figure 10 also represents topomaps generated for an Epileptic patient during a seizure, with the topomaps being spread over a total duration of approximately 95ms.

After processing and extracting the relevant features from the raw EEG data from both the categories—Seizure and Non-Seizure, the data was concatenated to form a single data Table for applying different kinds of Machine Learning classifiers. This work employed classifiers like Support Vector Machine, K-Nearest Neighbor Classifier and Random Forest Classifier to achieve the required classifications.

**Figure 11:**
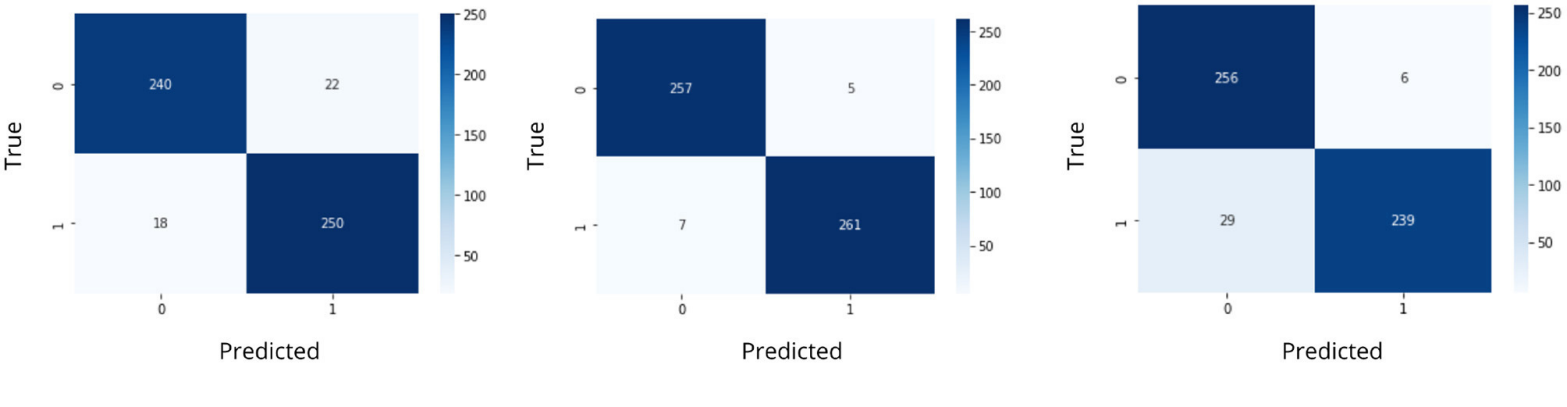
Confusion matrices using classifiers for iSeizdiag.

The data was split in the ratio of 70%-30% for generating training and testing data for feeding into the classifier. The models were trained and the confusion matrix and accuracy score were obtained. The confusion matrices for seizure detection using classifiers are represented in Figure 11.

**Figure 12:**
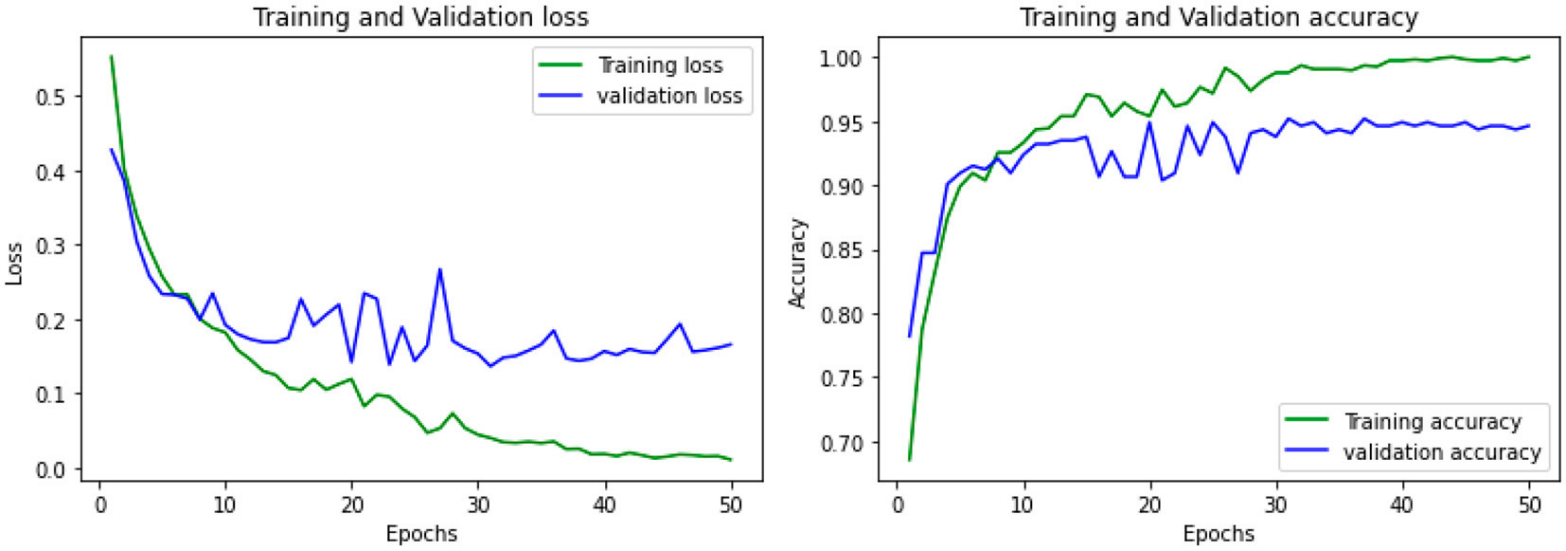
Accuracy analysis using ANN model using multiple epochs.

**Figure 13:**
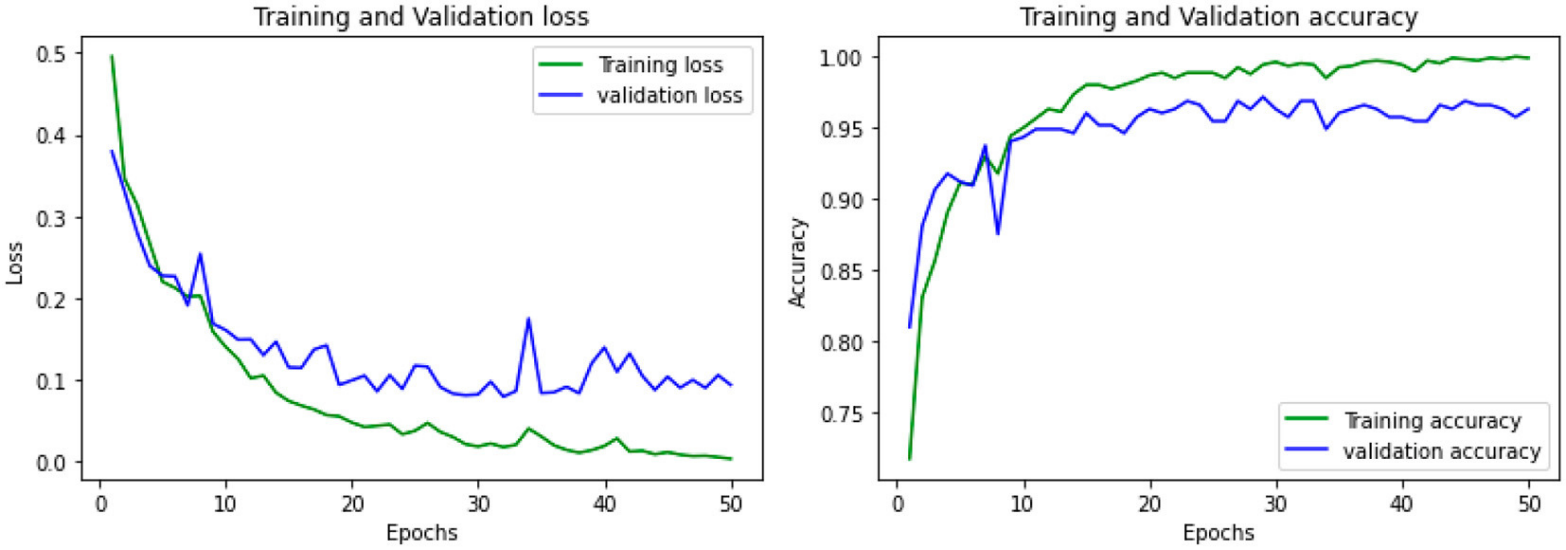
Accuracy analysis using CNN model using multiple epochs.

The accuracy was obtained for each of the classifiers for the EEG data of 5 patients. Support vector Machine performed with an accuracy, which is claimed in the range of 92.4%–95.2%. K-Nearest Neighbors Classifier and Random Forest Classifier outperformed the Support Vector Machine classifier with accuracy of 93.39% and 97.73% respectively. The detailed results involving the numbers from the confusion matrix and the accuracy for the respective algorithms are represented in Table 1. The ANN and CNN-based computing models represented the accuracy, which represents the better-performing model comparatively. The analysis is represented in Figures 12, 13. Some statistical parameters are also obtained to analyze the optimized model for seizure detection, which are represented in Table 2.

**Table 1:** Machine learning algorithms used.

**Algorithm used**	**TP**	**FP**	**FN**	**TN**	**Accuracy**
SVM model	237	20	21	252	92.2
KNN models	249	8	11	262	96.4
RF classifier	249	8	11	62	96.4

**Table 2:** Performance parameters using different models.

**Models**	**Precision**	**Recall**	**F1-score**	**Accuracy**
RBF	0.95491	0.875	0.913	0.916
RF	0.973	0.962	0.967	0.96
KNN	0.935	0.924	0.93	0.93
ANN	0.995	0.923	0.958	0.965
CNN	0.996	0.924	0.96	0.97

During DWT signal processing, the statistical parameters are calculated from discrete coefficients. The analytical results are tabulated in Table 3. The proposed framework for seizure and non-seizure detection is also compared with prior good work. The proposed work is represented in the context of quite the advancement of previous frameworks, which are represented in Table 4.

**Table 3:** Statistical parametric analysis for DWT.

**Coefficient**	**Mean**	**Deviation**	**Skew**	**RMS**	**Kurtosis**
1st	137.88	295.68	0.04	320.10	0.61
2nd	7.68	107.272	0.14	105.08	0.088
3rd	0.905	69.55	0.12	68.64	0.06
4th	2.76	19.51	0.05	19.56	0.23

**Table 4:** Comparison with previous work.

	**Lin et al. (2018)**	**Li et al. (2017)**	**Sayeed et al. (2019a)**	**Yan et al. (2022)**	**Proposed work**
Signal	Spectrum	Time	DWT	MS-	DWT
Transform	Power	Frequency	model	WTC	model
Proposed	LDA	RBF	DNN	CNN	KNN,
Classifier	model	model	model	model	RF & SVM
Claimed	92.68%	>95%	>95%	94%	>97%
Accuracy					
Pattern	-	-	-	Spectral-	Temporospatial
Used				Temporal	Mapping
Specific	Seizure	Image	Seizure	Seizure	Seizure & non
Use	detection	classification	detection	prediction	-seizure detection

## 8 Conclusion and future work

The proposed work demonstrated epileptic seizure detection using signal processing techniques like DWT and an optimized classifier as a computing model. DWT is highly effective in feature extraction. Undoubtedly, it represents an improvement in the accuracy of such applications by using better feature extraction techniques and advanced machine learning techniques like Neural Networks. The desired accuracy and statistical parameters have been achieved to prove a reliable framework for seizure detection. With access to data from EEG signal acquisition systems, applications can be developed that can detect the occurrence of seizure episodes in Epileptic patients using appropriate signal processing and machine learning techniques. Automatic seizure detection based on EEG data can prove to be more than 85% successful. Such a framework will help detect other brain-related diseases with the primary focus being on depression detection and classification. As the disease is an infamous and known for being notorious in detection or classification, it is required to focus on other aspects apart from mere disease detection. The course of plan for the projected target would be:

- Anomaly detection to find a differentiating case among the non-depressed humans,
- Mapping disease detection with Sentiment Analysis to learn emotional side effects of depression in the patients,
- Detection and classification of depression using only the emotional (or sentimental) factor,
- Mapping other physiological records of the patient such as EMG, GSR and so on, to make a better study of the effects of depression on a human in daily life.

With the help of the above steps, it is possible to create a framework that could be of great use in the field of healthcare. The present work motivates us to design wearable devices for such kinds of healthcare, which would be low cost, high speed and easy to handle solutions.

## Data Availability

The original contributions presented in the study are included in the article/supplementary material, further inquiries can be directed to the corresponding author.
